# An integrated computational biology approach defines the crucial role of TRIP13 in pancreatic cancer

**DOI:** 10.1016/j.csbj.2023.11.029

**Published:** 2023-11-17

**Authors:** Swati Dhasmana, Anupam Dhasmana, Stella Rios, Iris A. Enriquez-Perez, Sheema Khan, Farrukh Afaq, Shafiul Haque, Upender Manne, Murali M. Yallapu, Subhash C. Chauhan

**Affiliations:** a Department of Immunology and Microbiology, School of Medicine, University of Texas Rio Grande Valley, McAllen, USA; b South Texas Center of Excellence in Cancer Research, School of Medicine, University of Texas Rio Grande Valley, McAllen, USA; c Department of Pathology, University of Alabama at Birmingham (UAB), Birmingham, AL, USA; d O’Neal Comprehensive Cancer Center, UAB, Birmingham, AL, USA; e Research and Scientific Studies Unit, College of Nursing and Allied Health Sciences, Jazan University, Jazan, Saudi Arabia; f Gilbert and Rose-Marie Chagoury School of Medicine, Lebanese American University, Beirut, Lebanon; g Centre of Medical and Bio-Allied Health Sciences Research, Ajman University, Ajman, United Arab Emirates

**Keywords:** TRIP13, Pancreatic cancer, Early events of cancer, Integrative Biology, Transcriptomics, Computational biology

## Abstract

Pancreatic cancer (PanCa) is one of the most aggressive forms of cancer and its incidence rate is continuously increasing every year. It is expected that by 2030, PanCa will become the 2^nd^ leading cause of cancer-related deaths in the United States due to the lack of early diagnosis and extremely poor survival. Despite great advancements in biomedical research, there are very limited early diagnostic modalities available for the early detection of PanCa. Thus, understanding of disease biology and identification of newer diagnostic and therapeutic modalities are high priority. Herein, we have utilized high dimensional omics data along with some wet laboratory experiments to decipher the expression level of hormone receptor interactor 13 (TRIP13) in various pathological staging including functional enrichment analysis. The functional enrichment analyses specifically suggest that TRIP13 and its related oncogenic network genes are involved in very important patho-physiological pathways. These analyses are supported by qPCR, immunoblotting and IHC analysis. Based on our study we proposed TRIP13 as a novel molecular target for PanCa diagnosis and therapeutic interventions. Overall, we have demonstrated a crucial role of TRIP13 in pathogenic events and progression of PanCa through applied integrated computational biology approaches.

## Highlights

• Integrative computational biology techniques are holistic approaches in cancer discovery by exploring multiple levels of convolution in biological systems.
• Pancreatic cancer is expected that by 2030, it will become the 2^nd^ leading cause of cancer related deaths in the United States alone.
• For the first time, the role and patholgenic events of TRIP13 in PanCa have been discussed.
• This study elucidates the role of TRIP13 on molecular level, as a specific signal for early events of pancreatic cancer, enhancing patient prognosis, and boosting targeted therapies in clinical settings.
• This study has potential to enrich the existing biomarker panel for the early detection of pancreatic cancer.

## Graphical Abstract

**Figure:**
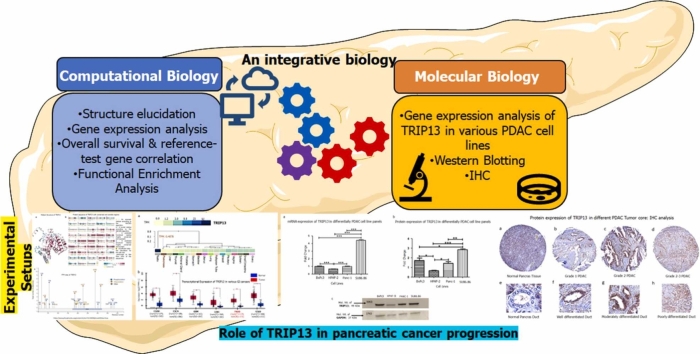

## 1 Introduction

Pancreatic cancer (PanCa) is one of the most aggressive forms of cancer, with very low 5-year survival and recovery rates for patients worldwide. In addition, the treatment efficacy of PanCa therapeutic regimens is not very good. Generally, pancreatic cancer is diagnosed at an advanced stage in > 80% of cases, which restricts the intervention of surgical and treatment opportunities. Recent epidemiological studies suggest that non-Hispanic Black males (17.6) and females (14.9) have highest incidence rate of PanCa as compared to American Indian (16.5:10.0), White (15.7:11.7), Hispanic (12.8:11.2), and Asian (11.0:9.2) counterparts [1]. Despite expansion of knowledge, we have experienced almost no or minimal progress in improving early diagnosis and treatment efficacy PanCa in the last 50 years.

At present, there are no standard diagnostic or validated early detection methods available for PanCa in public domain. Carbohydrate antigen 19-9 (CA19–9) is the only Food and Drug Administration (FDA) approved tumor specific biomarker for PanCa [2]. Despite the wide use of CA19–9 as a PanCa biomarker, it is an antigenic carbohydrate. CA19–9 evinces significant drawbacks in clinical applications such as being significantly more effective in identifying recurrence and evaluating response to augmentation treatment, and lacking sensitivity to detect pancreatic cancer in early stages. Only 65% of pancreatic cancer patients have elevated CA19–9 levels in their blood, meaning administering a CA19–9 test may provide false-negative results in these patients [3], [4], [5], [6]. Additionally, carcinoembryonic antigen (CEA), a glycoprotein expressed during fetal development is another common biomarker used for pancreatic cancer screening. In recent years, CEA has received attention as a diagnostic marker for several cancers such as colon cancer, stomach cancer, and lung cancer, however, its diagnostic potential remains elusive due to its poor sensitivity and specificity [7], [8], [9]. In the current scenario there is an urgent need to enrich the cancer biomarker panel to improve pancreatic cancer diagnosis, but these biomarkers should be specific to pancreatic cancer conditions. This specificity will be an added advantage if it only expresses in early stage and marks the presence in particular grade of cancer. In continuation to this investigation, we have recognized thyroid receptor interacting protein 13 (TRIP13) as a potential fit in all these criteria. As a member of the AAA+ ATPase enzymes (**A**TPase family **a**ssociated with various cellular **a**ctivities), TRIP13 has been linked to an array of cellular processes, including the checkpoint signaling, DNA break repair and recombination, and chromosome synapsis, amplified cell proliferation, tumor progression, and drug resistance in several different types of cancer tissues [10], [11]. The overall survival of multiple myeloma patients was poor with elevated TRIP13 expression [11] and aberrant TRIP13 expression in cancer cells leads to chromosomal recombination malformations [12]. Given the consequences of chromosomal defects in cell proliferation, TRIP13 overexpression can promote tumorigenesis by inducing chromosomal instability and aneuploidy [13]. The role of TRIP13 in prostate [14], colorectal [15], lung [16], liver [17], and multiple myeloma [18] has been identified, while its role in pancreatic cancer is not yet well defined.

In this study, the potential role of TRIP13 in pancreatic cancer is decoded by using an integrated computational biology and wet lab experimental approaches. Bioinformatics, datamining, transcriptomics, and molecular biology techniques were utilized to demonstrate the role of TRIP13 pancreatic cancer pathobiology and pancreatic cancer progression. Results of this study suggest that TRIP13 may be involved in early events of pancreatic cancer, thus, it might be a useful molecular signature for advancing early detection and therapeutics of pancreatic cancer.

## 2 Methods and materials

### 2.1 Structural interpretation of TRIP13

Various three-dimensional (3D) crystal structures of TRIP13 (Uniport ID: Q15645) are created using Research Collaboratory for Structural Bioinformatics (RCSB) Protein Data Bank. For better understanding and identification of highly conserved, variable, and functionally exposed residues of TRIP13, ConSurf server was used [19], [20], [21]. The algorithm of ConSurf is based on the separating functional residues of proteins by reviewing the grade of conservation of residues positions among their identical homologous protein sequence. Phosphosite was used to determine the location of various phosphorylation and ubiquitylation sites of TRIP13 (https://www.phosphosite.org/proteinAction?id=4465&showAllSites=true). The Human Protein Atlas was used for structural depiction, detailed fragments, unique and antigenic sequences of TRIP13 protein structure.

### 2.2 Normal and cancerous sites specific gene expression analysis of TRIP13, its correlation and survival analyses

The organ-specific gene expression coverage of TRIP13 in normal conditions was performed by utilizing Genotype-Tissue Expression (GTEx) server [22]. Whereas the comparative gene expression of TRIP13 in normal and gastro-intestinal (GI) conditions was performed using GEPIA2 [23]. The gene expressions were assessed on the scale of log2(TPM + 1). The total sample size of control and patients in various GI cancers were noticed as colon cancer (COAD: 275 *vs* control 349), esophagus cancer (ESCA 186 *vs* control 286), gallbladder cancer (GBM: 163 *vs* control 207), liver cancer (LIHC: 369 *vs* control 160) pancreatic cancer (PAAD: 179 *vs* control 171) and stomach cancer (STAD 408 *vs* control 211). GEPIA2 correlation analysis was performed using pairwise gene correlation between TRIP13, CEACAM5 and S100A4. The spearman correlation coefficient was applied to calculate the correlation. The Cancer Genome Atlas (TCGA) tumor, TCGA normal, and GTEx databases were used to get the correlation between all genes. The overall survival (OS) based on TRIP13 expression was performed by following Kaplan-Meier Plot from GEPIA2. Overall survival analysis median was the group cutoff of 95% of confidence interval (CI) and hazard ratio (HR more than 1).

### 2.3 Gene expression by pathological stage, isoforms, and location of TRIP13 in single cell type clusters of pancreatic cells

A stage plot diagram was used to showcase the expression of TRIP13 across PAAD stages (Stages I, II, II, and IV). The violin plot of gene expression division and isoform structure of TRIP13 in PAAD condition was measured by using isoform details sub-component of GEPIA2. In pathological staging the TRIP13 expression was documented on log2(TPM + 1) level. The cellular location of TRIP13 in pancreatic cell cluster was evaluated by scRNA-seq using UMAP plot, from the Protein Atlas database [24] based on healthy human tissues. Using this approach, the predictive positioning of TRIP13 in precise cell clusters of pancreatic cells was identified in the range of 0–30 nTPM.

### 2.4 PDAC cell line cultures

The commercially available Pancreatic Ductal Adenocarcinoma (PDAC) cell lines were acquired from American Type Culture Collection (ATCC, Manassas, VA, USA). For this study, differentiation grades of PDAC cell lines, like well differentiated cell line (HPAF-II), moderately differentiated cell line (BxPC3 & SU86.86), and poorly differentiated cell line (PANC1) were selected. The DMEM/F12 (Cat. No. 11320033, Gibco, for HAPH-II), RPMI-1640 (Cat. No. 11875–093, Gibco, for BxPC3 & SU86.86), and DMEM (Cat. No. 11965092 Gibco, for PANC1) media complemented with 10% fetal bovine serum (FBS) and 1% (w/v) penicillin–streptomycin was used for the cell cultivation (Gibco, ThermoFisher Scientific, Grand Island, NY, USA) under humidified atmosphere with 5% CO_2_ at 37 °C [25].

### 2.5 Gene expression analysis of TRIP13 in various PDAC cell lines

RNA was extracted from various PDAC cell lines using TRIzol (Invitrogen, USA). Reverse transcription was done using High-Capacity cDNA reverse transcriptase kit (ThermoFisher) as per manufacturer’s protocol. After collecting cDNA, it was amplified using TRIP13 and β-actin specific primers. Quantitative real-time PCR was performed using SSO Fast EvaGreen supermix (Bio-Rad). The relative expression levels of TRIP13 mRNA were assessed by Bio-Rad CFX96 using sequence-specific primers using previously described protocol [25]. The forward and reverse primers of TRIP13 and β-actin were ordered from IDT and sequences were TRIP13 forward primer 5’-ACT GTT GCA CTT CAC ATT TTC CA-3’; TRIP13 reverse primer: 5’-TCG AGG AGA TGG GAT TTG ACT-3’. β-actin forward primer: 5’-GTG CTA TCC CTG TAC GCC TC-3’; beta-actin reverse primer 5’-GAG GGC ATA CCC CTC GTA GA-3’.

### 2.6 Protein expression analysis

#### 2.6.1 Immunoblotting analysis of cell lines

TRIP13 antibody (Monoclonal mouse # OTI2F5, Cat No. CF809737, 1:1000; OriGene) was used as a primary antibody and GAPDH was used as endogenous control (anti-rabbit Cat:14C10 Cell Signaling) for the western blot as per previously published protocol [25]. Secondary antibody was procured from Promega (anti-mouse W4028).

#### 2.6.2 Human pancreatic tissues collection and Immunoblotting analysis

Human pancreatic ductal adenocarcinoma (PDAC) and their corresponding control tissue specimens were obtained from consented PDAC patients by the Anatomic Pathology Division of the University of Alabama at Birmingham (UAB). The UAB Institutional Review Board and Ethics Committee (IRB#060911009) has approved the utilization of control/tumor specimens. Frozen tissues were used for analysis of protein. The study was performed in accordance with the standards set by the Declaration of Helsinki. The TRIP13 protein expression in cells and in human samples was determined by western blotting. Briefly, protein lysates (30–50 µg protein) obtained from human samples were loaded onto NuPAGE™ 4–12% Bis-Tris Midi Protein Gels (Invitrogen, ThermoFisher Scientific, Carlsbad, CA, USA). The proteins from gels were transferred onto PVDF membranes (EMD Millipore, Billerica, MA, USA). The membranes were incubated with TR1P13 primary antibody (Catalog # 19602–1-AP, Proteintech, Rosemont, IL, USA) at 4 °C overnight, and then incubated with HRP-conjugated rabbit secondary antibody (Catalog # SA00001–2; Proteintech, Rosemont, IL, USA) for 1 h. Signals on the membranes were developed according to the manufacturer’s protocol (EMD Millipore, Billerica, MA, USA). The membrane was probed with β –actin (Catalog # HRP-60008; Proteintech, Rosemont, IL, USA) as loading control.

#### 2.6.3 Immunohistochemistry (IHC) analysis

Pancreatic carcinoma tissues microarray (TMAs, Cat no. PA2072B) containing cancerious and normal tissue cores were obtained from US Biolab Corporation Inc (Rockville, MD, USA). TRIP13 expression was assessed by immunohistochemistry (IHC) staining using a commercially available kit (BioCare Medical). The primary test antibody is TRIP13 antibody (Monoclonal mouse # OTI2F5, Cat No. CF809737, 1:800; OriGene) and IHC protocol was followed as per earlier published article [25]. After IHC procedure, the TMAs were digitally scanned and analyzed by 3DHISTECH Pannoramic MIDI (3D Histech) for TRIP13 staining.

### 2.7 TRIP13 associated co-expressed genes of and its functional enrichment analysis

LinkFinder (for co-expressed gene association) and LinkInterpreter (for functional enrichment analysis) sub-segments of LinkedOmics [21], [25], [26] were applied for identifying the genes that demonstrated disparity in association with TRIP13 in Pancreatic Cancer. For gene functional enrichment analysis Gene Set Enrichment Analysis (GSEA) database was used, under the KEGG Pathways plugin. The parameters for enrichment analysis were lowest no. of IDs in the 03 categories, highest no. of IDs in the 2000 categories, top 25 significance levels, 500 permutations.

### 2.8 Statistical analysis

For qPCR and western blot analyses, the statistical analyses were conducted applied unpaired, one-tailed Student’s t-tests. All error bars used in the attached graphs indicate the standard error of mean (SEM). The number of stars used on each graph signifies the level of significance – i.e., one-star (*) indicates P-values below 0.05, two stars (**) values below 0.01, and three stars (***) for values below 0.001.

## 3 Results

### 3.1 Structural interpretation of TRIP13

**Fig. 1:**
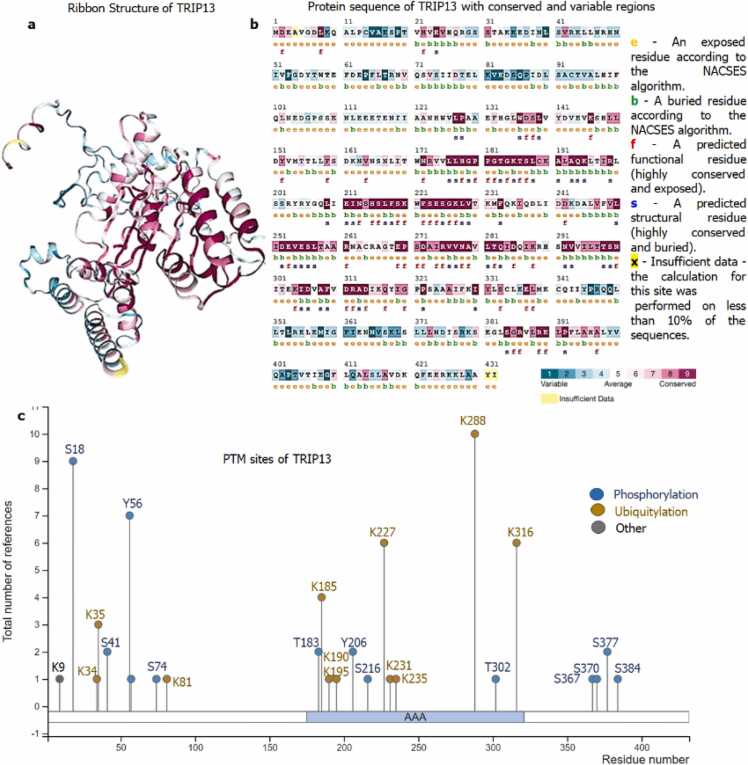
**Structural elucidation of TRIP13. a)** 3D model structure of TRIP13; **b)** Location of highest conserved (dark pink colored), exposed and functionally (notation by f) active residues of TRIP13; **c)** Positioning of phosphorylation and ubiquitylation sites in TRIP13. In this elucidation, TRIP13 structure was fragmented in various domains with exact location, like antigenic regions (31–148 & 219–324 aa), unique region (250–428 aa), P-loop containing nucleoside triphosphate hydrolase (110–335 & 135–400 aa), AAA+ ATPase domain (171–323 aa), ClpA/B family (179–193 & 289–303 aa) and ATPase, AAA-type, conserved site (293–312 aa).

**Fig. 2:**
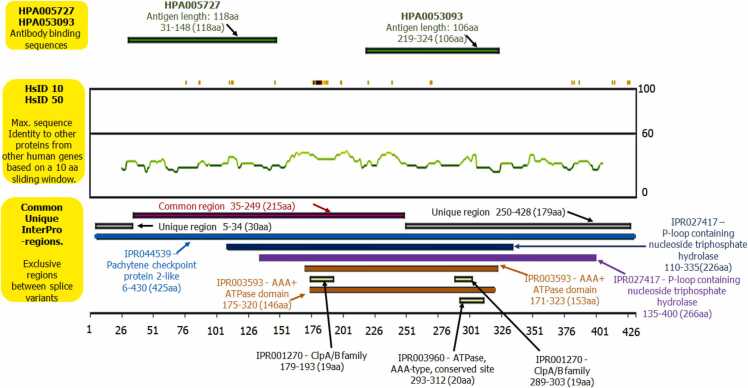
Structural explanation of numerous significant fragments, domains, and regions of TRIP13.

The protein sequence of TRIP13 was retrieved from uniport database (Uniport ID: Q15645). The retrieved sequence was considered as input query sequence for the ConSurf server. The input query created a 3D model of TRIP13 (Fig. 1a), based on created model the ConSurf server predicted 86 out of 432 amino acids as highest conserved and among 86 amino acids around 36 amino acids were exposed, and functional residues (Fig. 1b). Most of the highly conserved, exposed, and functional residues are in AAA+ ATPase domain (171–324 aa). TRIP13 also holds various phosphorylation (S18, S41, Y56, S74, T183, Y206, S216, T302, S367, S370, S37 & S384) and ubiquitylation sites (K34, K35, K81, K185, K190, K195, K227, K231, K235, K288 & K316) as shown in Fig. 1c. The structure of TRIP13 was minutely scrutinized by the Human Protein Atlas for the better elucidation of various important domains, regions, and fragments (Fig. 2).

### 3.2 Differential expression levels of TRIP13 in different types of normal and cancer tissues, its correlation and survival analyses

**Fig. 3:**
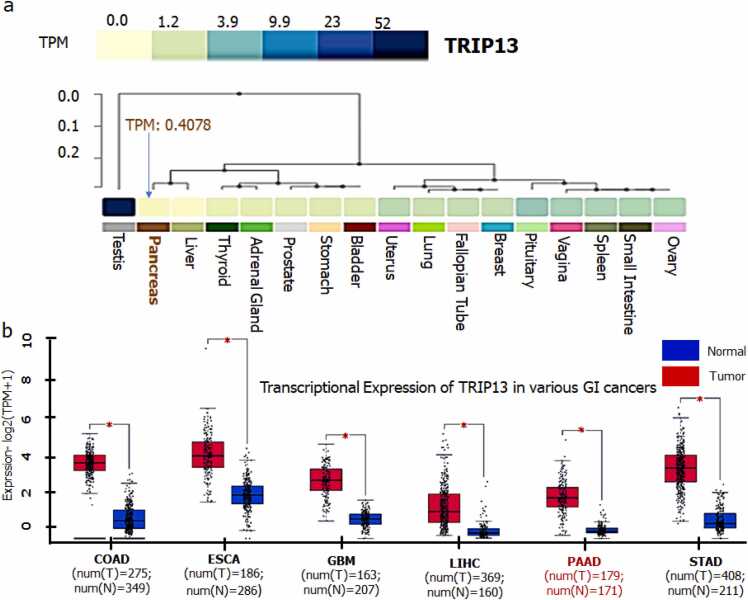
**Expression profiling of TRIP13 in normal and cancerous condition. a) Expression analysis of TRIP13 in normal condition:** GTEx server-based comparison of the mRNA expression level of test gene TRIP13 in normal and diseased conditions. The expression level of TRIP13 in normal condition is very less, 0.4078 TPM. **b) Differential gene expression box plot analysis of TRIP13 in various GI cancers:** GEPIA box plot analysis shows significantly higher mRNA expression of TRIP13 in PAAD followed by LIHC, while in normal condition the expression is almost negligible. The gene expression profile across all tumor samples and paired normal tissues was depicted on a dot plot. Each dot represents the expression of a sample. The expression data were scaled by log_2_(TPM+1) transformed for differential analysis and Log2FC Cutoff 1, is defined as median (Tumor) – median (Normal), p-value Cutoff is 0.01. Genes with higher log 2FC values and lower q values than pre-set thresholds are considered differentially expressed genes. The red colored box is tumor tissue dataset and blue colored box is normal tissue dataset. Red * denoted significant data.

GTEx server was applied to evaluate the mRNA expression level of TRIP13 in normal/disease free conditions. Among various organs, normal pancreas was expected to compare TRIP13 gene expression with several normal organs. In this analysis TRIP13 showed an almost negligible (0.4078 TPM) expression in pancreas followed by liver (Fig. 3a), while rest other organs had relatively higher expression of TRIP13 in normal condition. To validate differential expression of TRIP13, in important GI cancers (COAD, ESCA, GBM, LIHC, PAAD, and STAD), box plot analysis was performed, where we have noticed that PAAD and LIHC were only cancer conditions, that showed remarkably higher TRIP13 expression. Whereas in other cancers (COAD, ESCA, GBM, and STAD) there was no encouraging expression pattern of TRIP13 as compared to their respective normal tissues (Fig. 3b).

**Fig. 4:**
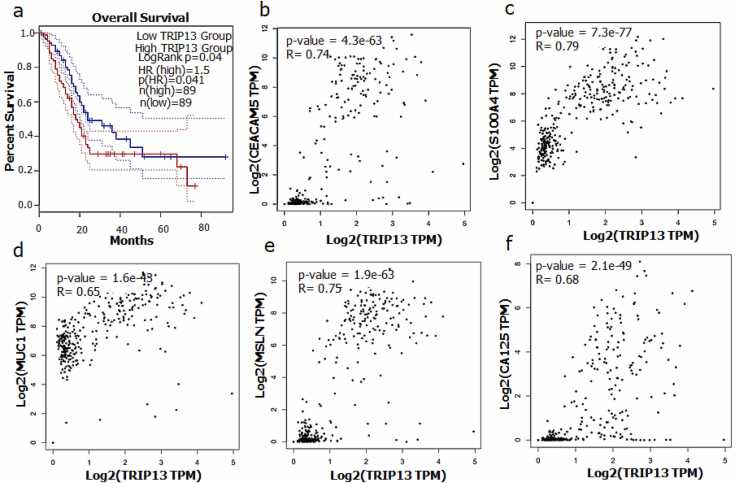
**Patients’ survival and gene correlation plots of TRIP13**. **a)** This survival plot was generated by GEPIA Kaplan-Meier Plot. GEPIA uses Log-rank test, also known as the Mantel–Cox test, for hypothesis test. The cox proportional hazard ratio and the 95% confidence interval (CI) information was also included in the survival plot. (Hazard Ratio (HR): 1.5, Logrank p = 0.04. This analysis was done based on mRNA expression. It can be observed from the Kaplan-Meier graph that less than a period of 80 months; patients with a low expression of TRIP13 have almost more than 100 months survival. **b-f)** Correlation graphs of TRIP13 with other biomarkers of pancreatic cancer like CEACAM5, S1004A, MUC1, MSLN and CA125 which demonstrate strong positive association of all genes in PAAD. The pair-wise gene expression correlation analysis of TCGA and/or GTEx expression data set was used with spearman correlation coefficient method.

Kaplan-Meier Plot (Hazard Ratio (HR) 1.5, Logrank p = 0.04 and sample size 178) was utilized to determine the relationship between overall survival and TRIP13 expression in PAAD. In this analysis, higher TRIP13 expression was correlated with shorter overall survival (less than 80 months) in PAAD patients, while lower expression showed relatively better survival (almost 100 months or more) (Fig. 4a). Additionally, a correlation analysis was performed to test the significance of test gene (TRIP13) in PAAD in relation to some of the other reference genes (CEACAM5, S100A4, MUC1, MSLN and CA125). The final R and p values were 0.74, 0.79, 0.65, 0.75 and 0.68 (strong positive association) and p value 4.3e-63, 7.3e-77, 1.6e-43, 1.9e-63 and 2.1e-49, respectively (Fig. 4b, c, d, e & f).

### 3.3 Gene expression by pathological stage, isoforms, and location of TRIP13 in single cell type clusters of pancreatic cells

**Fig. 5:**
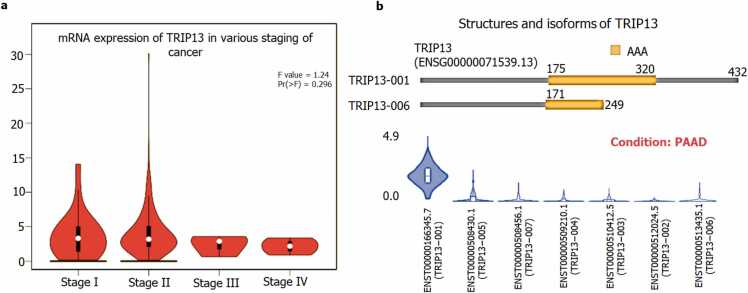
**Pathological staging and isoforms of TRIP13**. **a) Pathological staging of TRIP13:** The mRNA expression of TRIP13 in various stages of pancreatic cancer (stage I to IV). The expression data are first log2(TPM+1) transformed for differential analysis. The method for differential gene expression analysis is one-way ANOVA, using pathological stage as variable for calculating differential expression. **b) Structure and Isoforms:** Protein structural features of long and short form of TRIP13 and 07 TRIP13 transcripts in PAAD condition. Only two transcripts ENST00000166345.7/Uniprot ID: Q15645 (TRIP13 −001) and ENST00000513435.1/Uniprot ID: H0YAL2 (TRIP13 −006) are protein coding isoforms of TRIP13.

**Fig. 6:**
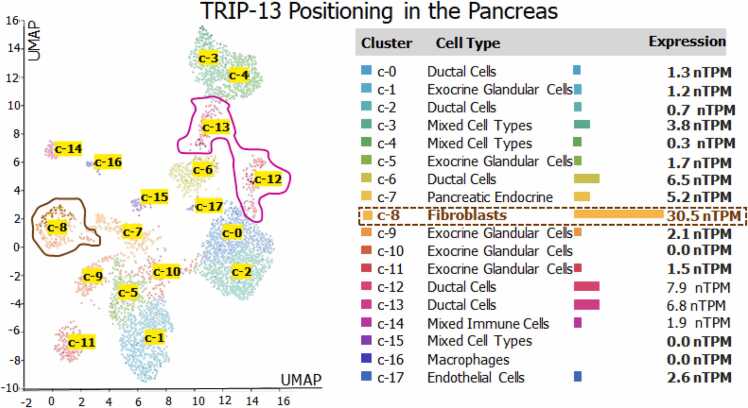
**TRIP13 expression positioning in different pancreatic cell types.** RNA expression of TRIP13 in different compartments of pancreas tissues. The single cell type clusters identified in pancreatic tissue were visualized by a UMAP plot.

The initial staging of PAAD observed very high mRNA expression of TRIP13, especially in stage I and II, while stages III- IV have shown relatively lower expression (Fig. 5a). A total of 07 transcripts of TRIP13 were recognized in the isoform analysis. ENST00000166345.7 (TRIP13 −001) and ENST00000513435.1 (TRIP13 −006) are protein coding transcripts, and remaining ENST00000508430.1 (TRIP13 −005); ENST00000508456.1 (TRIP13 −007); ENST00000509210.1 (TRIP13 −004); ENST00000510412.5 (TRIP13 −003), and ENST00000512024.5 (TRIP13 −002) are intronic or nonsense transcripts (Fig. 5b). Among all 07 transcript only 02 transcripts were protein coding transcripts those were liable for synthesis of long form of protein with 432 aa (Isoform ID: ENST00000166345.7 and Isoform symbol: TRIP13–001), this form has bigger segment of AAA domain with 145 aa from 175 to 320, while short form of protein consists total 249 aa (Isoform ID: ENST00000513435.1 and Isoform symbol: TRIP13–006) of TRIP13. The shorter form of TRIP13 consists of 78 aa length of AAA domain which starts from 171 to 249 as shown in Fig. 5b. The apparent site of TRIP13 expression in the pancreatic cell clusters are plotted based on a UMAP plot (Fig. 6). The maximum expression was noticed in the fibroblast cells cluster followed by ductal cells, pancreatic endocrine cells, mixed cell types and endothelial cells. These single cell type clusters are indicating the higher expression of TRIP13 in fibroblast cells like C-8 (30.5nTPM) and ductal cell clusters like C0, 2, 6, 12 and 13 (1.3, 0.7, 6.5, 7.9 and 6.8 nTPM).

### 3.4 TRIP13 expression analysis in PDAC cell lines and TMAs

**Fig. 7:**
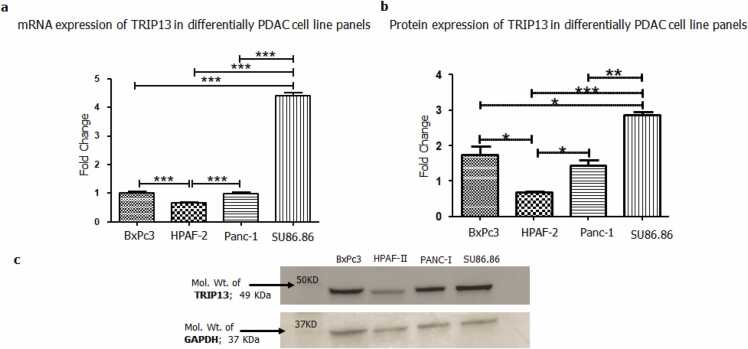
**Expression of TRIP13 in PAAD/PDAC cell lines. a)** mRNA expression analysis: Gene expression of TRIP13 in progressive PDAC cell lines n = 3; p-value * <= 0.05 and * ** <= 0.001. **b)** Protein expression: Protein expression of TRIP13 in progressive PDAC cell lines model was determined using western blot. **c)** Representative image of western blot of TRIP13. (n = 3); p-value * <= 0.05, * * < =0.01 and * ** <= 0.001.

qPCR analysis was performed to investigate the mRNA expression level of TRIP13 in various PDAC cell lines like HPAF-II, BxPC3, SU86.86, and PANC1 that represent differentiation stages (well, moderately and poorly differentiated). This fold change observation provides an interesting trend of TRIP13 expression in this cell line model system. SU86.86 showed the highest mRNA expression of TRIP13, followed by BxPC3; PANC1 and HPAF-II cells (Fig. 7a). To determine the expression at protein level, western blot analysis was performed which validated the similar expression pattern of TRIP13 in these PDAC cell lines (Fig. 7b & c).

**Fig. 8:**
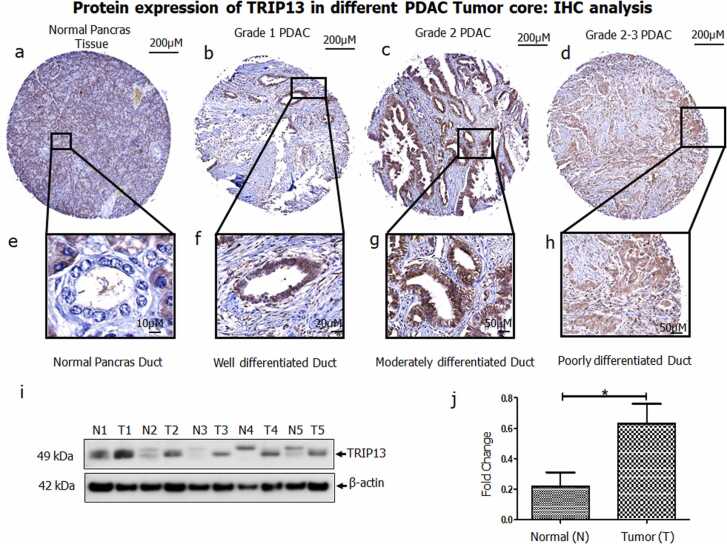
**The representative IHC images of pancreatic adenocarcinoma tissues cores. a&e)** Normal pancreatic tissues and ducts; **b&f)** Grade 1 malignant pancreatic adenocarcinoma; **c&g)** Grade 2 malignant adenocarcinoma; **d&h)** Grade /3 pancreatic adenocarcinoma. **i)** Increased expression of TRIP13 in human pancreatic ductal adenocarcinomas (PDACs). Western blot analysis showing expression of TRIP13 in adjacent normal pancreas (N) and PDAC (T) human tissue samples. The N and T corresponding samples were obtained from patients with PDAC. **j)** Densitometry graph of protein expression of TRIP13 in human adjacent normal tissues and PDAC.

IHC analysis on human PDAC TMAs tumor cores followed the similar trend of TRIP13 expression (Fig. 8). The expression of TRIP13 in representative cores of normal pancreatic tissues, grade 1 (well differentiated), 2 (moderately differentiated) and 2–3 (moderately to poorly differentiated) samples is shown in Fig. 8(a-h). For clarity we have also provided zoomed images of all groups. It is evident that normal duct is unreactive with TRIP13 antibodies while grade 2 spots demonstrated higher intensity as compared to grade 1 and grade 3. The western blot analysis clearly depicted these human adjacent normal tissues have less expression of TRIP13 as compared to pancreatic cancer tissue samples as shown in Fig. 8i & j.

### 3.5 TRIP13 associated co-expressed genes and their functional enrichment analysis

**Fig. 9:**
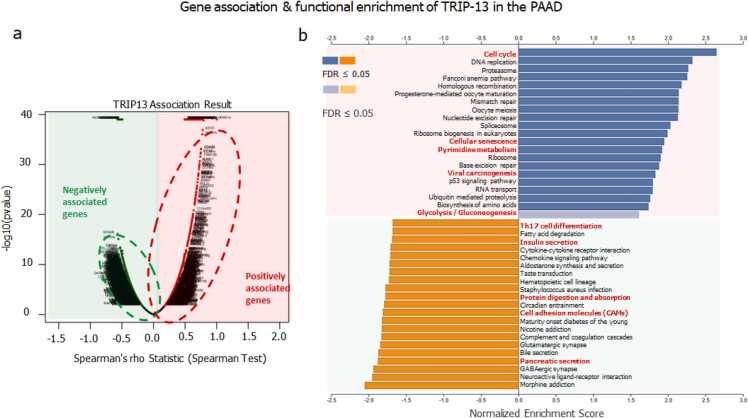
**Volcano graph and pathways enrichment analyses of TRIP13 and associated genes. a)** Co-expressed genes of TRIP13 in pancreatic cancers. The significantly correlated genes with TRIP13 were analyzed by Spearman test. Green and red colored partition represented the negative and positive correlated genes with TRIP13, respectively. **b)** Functional enrichment analysis of TRIP13 and associated genes. Blue and orange bar graphs represent pathway enrichment of positively and negatively associated genes with TRIP13, respectively.

To explore the prospective impact of TRIP13 on various pathways and its associated genes; TCGA_PAAD search, and target dataset of UNC institute were selected for the RNAseq analysis using HiSeq RNA platform and Firehose_RSEM_log2 pipeline. The patient sample size was 178. In this enrichment analysis a total of 11,428 genes exhibited negative correlations (green dots) with TRIP13, and the remaining 8346 genes displayed positive correlation (red dots) with TRIP13 (Fig. 9a). For functional and pathways enrichment of the co-expressing genes with TRIP13, enrichment explores using GSEA and KEGG pipelines in the LinkInterpreter platform. As demonstrated in Fig. 9b, the TRIP13 co-expression genes have positive substantial associations in DNA repair machinery like DNA replication, Fanconi anemia pathway, homologous recombination, mismatch repair, nucleotide excision repair, and base excision repair. Apart from that, other physiological functions include cellular senescence, viral carcinogenesis, and p53 signaling pathway. Whereas Th17 cell differentiation, insulin secretion, protein digestion and absorption, cell adhesion molecules (CAMs), pancreatic secretion, bile secretion showed negative correlation with TRIP13.

## 4 Discussion

Pancreatic cancer (PanCa) is an ominous health concern, and it will become the second leading cause of cancer related deaths in the United States by 2030. Still health professionals are struggling to diagnose PanCa at early stages of the disease condition. PanCa is typically detected at a late stage, with extremely low survival and substantial metastases rate [27]. The existing PanCa diagnosis panel is very conventional (CA19–9, CA125, MUC1 and carcinoembryonic antigen/CEA) [21], [25], [28], [29], and less precise to detect PanCa in early stages. Concerning this clinical unmet need, our group has explored the link between PanCa and TRIP13. TRIP13 (Thyroid Hormone Receptor Interacting Protein 13) is one of AAA (**A**TPase family **a**ssociated with various cellular **a**ctivities) protein, which is involved in various cellular processes, including the checkpoint signaling, DNA break repair-recombination, and chromosome synapsis [10]. Current study has tried to uncover the salient features of TRIP13 by operating an integrated computational approach. Core streams like bioinformatics and molecular biology techniques were utilized to define the role of TRIP13 in PanCa. The protein structural features of TRIP13 were systematically conferred like most of functionally exposed residues and phosphorylation sites were situated in AAA domain of TRIP13, which explicitly accountable for TRIP13 associated cellular activities. The descriptive features of TRIP13 like antibody binding sites (31–148 & 219–324 aa), unique regions (5–34 & 250–428 aa), pachytene checkpoint (6–430 aa), p-loop containing NTP hydrolase (110–335 aa), AAA+ domain (171–323 aa) and so many other important sites were identified in this investigation. TRIP13 is identified as a tumor associated protein in various tumors but its function in oncogenesis is still not clear. Previous studies however, state that, the AAA+ ATPase domain of over expressed TRIP13 is responsible for the unfolding of proteins to modify their activity. TRIP13 has affinity to interact an adaptor protein, trailed by this dimer complex permits it to connect to the closed form of MAD2 and this interaction start the unfolding of MAD2 followed by turning off the spindle assembly checkpoint and safety belt disengagement. This whole process leads to the various mutations which may be responsible for the oncogenesis [30]. The transcript per million (TPM) expression of query gene was listed in various organs at normal and diseased states. Surprisingly TRIP13 expression was almost negligible in normal pancreas as compared to other organs like testis, uterus, lungs, breast, vagina, spleen, intestine, and ovary. While in PanCa state logTPM value of TRIP13 is significantly higher, it seems like almost 6 times higher than normal pancreas. This study indicates that higher expression of TRIP13 is linked to poor patient’s survival. For correlation analysis CEACAM5, S100A4, MSLN, MUC1 and CA125 were used as reference genes against TRIP13 for PanCa condition [31], [32], [33], [34] [21]. In this investigation, all these reference genes have shown strong and positive correlation with TRIP13. The long protein coding isoform of TRIP13 (ENST00000166345.7 and total 432 aa) was found to be significantly over expressed in PanCa condition, as compared to short protein coding isoform (ENST0000513435.1 and total 249 aa). TRIP13 expression analysis in the various pathological staging demonstrates relatively higher expression of TRIP13 in early stages like I and II (II stage was showing higher expression than stage I), as compared to the advance stages (III-IV) of PanCa. This relation strongly suggests the involvement of TRIP13 in early events of PanCa. The protein atlas based single cell seq analysis demonstrates the expression of TRIP13 in fibroblast and ductal cells. As we know fibroblasts are a crucial component of the stroma and these fibroblasts also interact with cancerous cells through various trails like transforming of extra cellular matrix (ECM), reprogramming of metabolic activities, invasion, metastasis, and drug resistance activities [35]. Pancreatic ductal adenocarcinoma (PDAC) contributes around 85–90% of PanCa [25]. Thus, TRIP13 can be a crucial player of tumor microenvironment and tumorigenesis.

In sequel of integration approach, molecular biology techniques were applied to validate bioinformatics data. The progressive PDAC cell lines were used to identify the mRNA and protein expression level of TRIP13. An almost similar trend was observed in qPCR and western blot experiments, like moderately differentiated cell lines SU86.86 and BxPC3 showed higher expression while HPAF-II and PANC1 showed relatively lower expression. Additionally, TMA expression analysis suggests higher expression of TRIP13 in stage II, as compared to other stages, supporting the *in-silico* datamining evidence. The human tumor cores also confirmed immuno-reactivity with the TRIP13 MAb. Especially moderately differentiated core was showing high intensity and higher coverage area while well differentiated and moderately to poorly differentiated cores were showing relatively lesser reactivity. After minute scrutinization of IHC results in various tumor cores, it notifies that the expression of TRIP13 was commonly found in cytoplasm and on the membrane and sometimes in the nucleus. The normal pancreatic duct was unstained and non-reactive for TRIP13 MAbs, suggests no or undetectable level of TRIP13 in normal pancreas.

The functional and pathways enrichment analysis explored that TRIP13, along with its co-expressed genes significantly contributes to the normal physiological, pathological, and metabolic functions. Positively corelated genes are involved in several pathways like cellular senescence: although this function is related to tumor suppression but it can be inappropriate if senescent cells stay continuously in transformed tissues, which may trigger a surfeit of tumor‐promoting factors [36]. Viral carcinogenesis is the one of the most common cause of carcinogenesis where several tumor viruses (Human papilloma virus; Hepatitis B virus; Hepatitis C virus; Epstein–Barr virus; Kaposi sarcoma herpesvirus; Merkel cell polyoma virus; Human T-cell lymphotropic virus, type-1; human cytomegalovirus; human herpesvirus-6 and adeno-associated virus-2) are involved in tumorigenesis by activating insertional mutagenesis, viral oncogenes, and immunosuppression processes [37]. Glycolysis/gluconeogenesis: tumor exhibit an elevated level of glycolysis in the presence of oxygen to boost cellular proliferation [38], whereas gluconeogenesis is a reverse pathway of glycolysis, which uses lactate or amino acids to feed biosynthetic pathways branching from glycolysis [39]; pyrimidine metabolism pathway: is actively involved in cancer proliferation thus depleting of pyrimidine ribonucleotide pools has long been believed an important possibility for cancer management [40] and various other pathways and machineries were listed. On the other hand, the negatively associated genes were involved in insulin and pancreatic secretions that can hamper the normal physiology of pancreas leading to several chronic diseases like pancreatitis, diabetes, and pancreatic cancer. Th17 cell differentiation: Th17 cells are recognized as efficient CD4 + T cells that perform a fundamental role in autoimmune diseases and inflammation which may be linked with anti-tumor responses [41], the depletion of this factor may nurture the favorable tumor environment. Another important pathway is cell adhesion molecules and depletion in adhesion tendency may lead the tumor metastasis [25].

Integrative biology is a multidisciplinary tactic to blend a complicated biological dataset with genomic, proteomics, and molecular biology, techniques. The major benefit of integrative biology lies in its holistic and comprehensive viewpoint, which leads to better understanding of complex biological systems and explaining biological problems through systematic approach. Integrative biology still needs to develop in the form of complexity and challenges in data integration. This field is in a developing phase and facing some problems like the knowledge gaps, deficiencies of trained human-data resources and last hostility to interdisciplinary collaboration between software developer and scientists. This investigation provides strong evidence that TRIP13 is expressing and contributing to the early events of PanCa. However, studies in a larger patient cohort and in-depth basic mechanistic studies will provide next level information to further understand the biological implications of TRIP13 in pancreatic cancer and this study will pave a strong pathway for future studies on TRIP13.

## 5 Conclusion

PanCa has extremely low survival and extensively high metastases rate. The existing PanCa diagnosis panel is very conventional, and less precise to detect PanCa in early stages. Concerning this inflammable issue, our group has explored the link between PanCa and TRIP13 using the integrated computational biology approach. Integrative biology techniques are holistic approaches in cancer biomarker discovery by exploring multiple levels of convolution in biological systems. In this study we have utilized integration of high dimensional omics data into low throughput wet laboratory experimentations to decipher the expression level of TRIP13 in various pathological staging and functional enrichment analysis. This study elucidates the role of TRIP13 on molecular level like functional enrichment analysis specifically suggesting that TRIP13 and associated genes involved in very important physiological pathways. Other wet lab experimentations demonstrate the expression of TRIP13 as a specific signal for early events of pancreatic cancer, this proper positioning may enhance patient prognosis, and boosting targeted therapies in clinical settings. This study has potential to enrich the existing biomarker panel for the early detection of pancreatic cancer. Furthermore, the integrative biology approach grants a golden opportunity to software-data engineers to team up with the basic-medical scientist to unpuzzle the complicated mysteries of biological sciences.

## Ethical statement

Human pancreatic ductal adenocarcinoma (PDAC) and their corresponding control adjacent normal tissue specimens for western blotting analysis were obtained from consented PDAC patients by the Anatomic Pathology Division of the University of Alabama at Birmingham (UAB). The UAB Institutional Review Board and Ethics Committee (IRB#060911009) has approved the utilization of control/tumor specimens. Remaining other pancreatic carcinoma cell lines (BxPC3, HPAF-II, SU86.86 and PANC1) and tissues microarray with normal tissues (TMAs Cat no. PA2072B) were purchased from ATCC and US Biolab Corporation Inc (Rockville, MD, USA), so no ethical approval was necessary for these studies.

## CRediT authorship contribution statement

Swati Dhasmana**:** Conceptualization, Methodology Software, Validation, Formal analysis, Investigation, Resources, Data curation, Writing - original draft, Writing - review & editing. Anupam Dhasmana**:** Conceptualization, Methodology, Software, Validation, Formal analysis, Investigation, Resources, Data curation, Writing - Original Draft, Writing - review & editing. Stella Rios**:** Methodology, Cell culturing, Software, Validation, Formal analysis, Investigation, Data curation.**.** Iris A. Enriquez-Perez**:** Methodology (IHC)**.** Sheema Khan**:** Methodology, Software, Validation, Formal analysis, Writing - Review & Editing**.** Farrukh Afaq**:** Tissue Resource, Methodology, Formal analysis**.** Shafiul Haque**:** Methodology, Software, Validation, Formal analysis, Writing - Review & Editing**.** Upender Manne**:** Methodology, Software, Validation, Formal analysis, Review & Editing.**.** Murali M Yallapu**:** Supervision, Project administration, Writing - review & editing, Visualization, Funding acquisition. Subhash C Chauhan**:** Conceptualization, Methodology, Investigation, Resources, Writing - original draft, Writing - review & editing, Visualization, Supervision, Project administration, Funding acquisition.

## Declaration of Competing Interest

The authors declare that they have no known competing financial interests or personal relationships that could have appeared to influence the work reported in this paper.
